# Comparative *in vitro* study of two methods for gingival biotype assessment 

**DOI:** 10.4317/jced.55049

**Published:** 2018-09-01

**Authors:** Leticia Sala, Raquel Alonso-Pérez, Ruben Agustin-Panadero, Alberto Ferreiroa, Ana Carrillo-de-Albornoz

**Affiliations:** 1 Professor, Department of Periodontology, School of Dentistry, Mississippi Institution of Madrid. Spain; 2 Researcher, Department of Prosthodontics. School of Dentistry, Complutense University of Madrid. Spain; 3 Adjunct professor, Department of Stomatology, Faculty of Medicine and Dentistry, University of Valencia. Spain; 4 Associate Professor, Department of Restorative Dentistry and Buccofacial Protheses. School of Dentistry, Complutense University of Madrid. Spain; 5 Associate Professor, Department of Periodontology. School of Dentistry, Complutense University of Madrid. Spain

## Abstract

**Background:**

The gingival thickness seems to have an important role in different dental treatments. There are different methods of quantifying this thickness, but it is not known which of them can be the most effective. The objective to assess the accuracy of two different methods for gingival thickness measurement: the transgingival needle probing (TGNP) and the tension-free caliper (TFC) in an *in vitro* model, by comparing them with direct physical measurements (reference standard).

**Material and Methods:**

Gingival thickness (GT) was evaluated in 27 female pigs with four implant sites 1, 2 and 3mm from the gingival margin with three different methods: 1) transgingival needle probing 2) tension-free caliper and 3) Direct visualization after making a incision in the mucosa and measuring GT with a periodontal probe. Wilcoxon test for paired samples were used with a confident level of 95%.

**Results:**

A total of 324 points were measured, 59% of the sites presented a thin biotype with DV, it was correctly assessed with the TGNP in 84% of the times and in 86% with the TFC. 41% of the sample presented thick biotype, 76% was the percentage measured with the TGNP and 0% of the sites evaluated with TFC resulted in this biotype.

**Conclusions:**

Transgingival needle probing constitutes an accurate method when measuring GT at different levels. Tension free caliper is not a good tool for assessing the gingival biotype as long as it is unable to predict thick biotype.

** Key words:**Periodontal Biotype, Gingival Thickness, Periodontal Tissue and Diagnosis.

## Introduction

Recently scientific interest has focused to determine the influence of gingival biotypes on dental treatments. Gingival biotype is described as the thickness of the gingiva in the faciopalatal dimension (1). It has demonstrated to exhibit a significant impact on the outcome of restorative therapy (2–4). The influence of the gingival thickness seems to be an important factor to take into account in the diagnosis of dental treatment as it plays an important role in wound healing in regenerative surgical procedures (1). It can also prevent complications due to orthodontic treatments (5) and helps to achieve predictable and esthetic outcomes in implantology (6–8). A direct correlation has been established between gingival biotype and its susceptibility to suffer recession after surgical and restorative procedures being the thin one the most susceptible to this phenomenon(9,10). Moreover, it has been documented in literature greater mean bone loss occurring around implants in sites with thin biotype compared to thick overlying mucosa (8).

Hence, a proper diagnosis of the periodontal biotype seems to be of great interest in order to take decisions (11) in implant dentistry. It can also be a tool for clinicians as they can quantify and monitor gingival changes through the treatment (12,13).

Claffey and Shanley (14) defined the thin tissue biotype as a gingival thickness < 1.5 mm, while the thick tissue biotype was referred as tissue thickness ≥ 2mm. Several methods have been proposed to measure gingival thickness:1) invasive methods such as transgingival probing (TRAN) (15) or the use of an endodontic file (15–17); 2) non-invasive techniques such as probe transparency through the free gingiva (7,18), plaster models(19), ultrasonic devices (20,21), the modified caliper (6,22), and most recently the Cone-Beam Computed Tomography (CBCT) (17,23) and the “puffed cheek” method (computed tomography scans with distended cheeks) (24).

The validity of ultrasound devices and computed tomography methods have been widely studied (17,25,26). However, the most frequently used method is the one that measure the visibility of the instrument when probing (7,18). The use of a tension free caliper to perform a direct measurement has also been used for many authors, even though it cannot be used for pretreatment evaluation (6,22), it can be useful for measuring the gingival thickness on implants previous to the prosthetic treatment or after tooth extraction. In general, methods currently used to discriminate thin from thick gingiva have shown limited reliability and accuracy (27).

This lack of accuracy, described as the amount of agreement between the information from the test under evaluation and the reference standard, in the methods and indices to evaluate the soft tissues make difficult to establish a definition of the gingival esthetic parameters in relation to successful implant restorations(10).

This *in vitro* study has the outcome to assess the accuracy of two different methods for gingival thickness measurement: the transgingival needle probing and the tension free-caliper in an *in vitro* model, by comparing them with direct physical measurements (reference standard).

## Material and Methods

An *in vitro* study was conducted in fresh mandibles of female pigs to evaluate the gingival thickness (GT). This study is reported according to the Standards for Reporting of Diagnostic Accuracy (STARD) (28).

-Sample size calculation

The sample size estimation was calculated for √=0.05 and a power (1-ß) of 80%. A variability of 0.5 ± 0.2 mm was considered to be clinically relevant based on the results of previous studies (13). The sample size equaled 11 subjects but it was increased to 27 for robust data achievement.

-Experimental animals

Female pigs aged between 1 and 1.5 years were controlled for diet, temperature, and light exposure under Spanish standards for animal care before their sacrifice. The farms supplying the animals were organized in accordance with EU and Spanish legislation against cruelty to animals. The study protocol of the study was approved by the Medical Ethics Commission of the University of Alcalá de Henares (Madrid, Spain).

The following inclusion criteria were adopted: fresh mandible less than 24 h after the death of the animal; presence of edentulous sites and adjacent teeth with similar gingival architecture in optimal conditions; and study areas with at least 4 mm of keratinized mucosa. Animals were recruited consecutively from the farm, and they were discarded if they did not fulfill the inclusion criteria. Each animal provided four implant sites, and on each one GT was measured at 1, 2 and 3 mm from the gingival margin, providing a totally of 324 study areas. Two animals were studied per day, and all measurements were done after a 6 months period.

-Data collection

**Figure 1:**
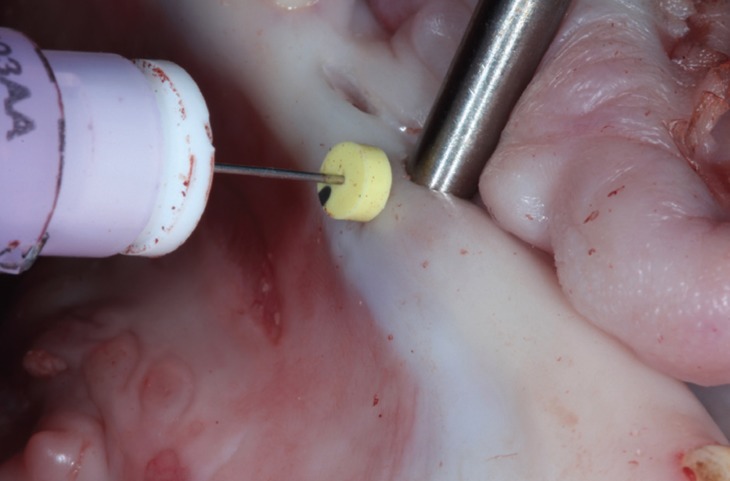
Transgingival needle probing (TGNP). Needle through the mucosa with a rubber stopper.

**Figure 2:**
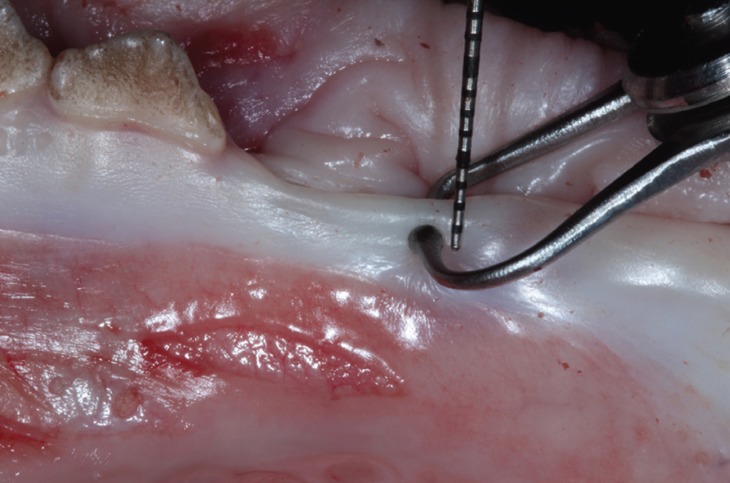
Tension-free caliper. Caliper measuring GT at 2 mm from the gingival margin.

**Figure 3:**
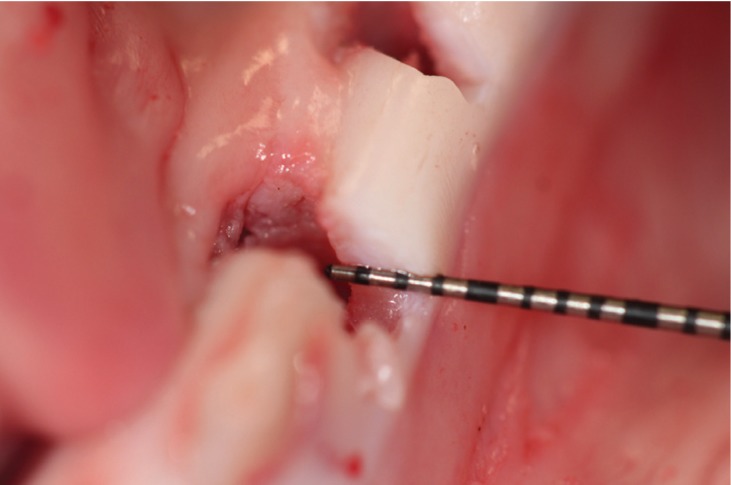
Direct visualization enables a direct measurement with a millimeter probe.

GT was evaluated by three different methods by the same operator (L.S.):

1) Transgingival needle probing (TGNP). An anesthesic needle was fitted with a rubber stopper (Normon Jet Plus 0.3x12 mm, Normon, Tres Cantos, Madrid, Spain). Previously, an abutment was placed in the implant (3inOne PYREA; 3.5 mm regular emergence profile, Biohorizons, Birmingham, AL, USA). The needle was placed perpendicularly into the mucosa in the points marked 1, 2 and 3mm apical to the gingival margin. The rubber stopper shifted along the needle while it went through the soft tissue until the abutment surface was reached. The distance between the needle tip and the silicone stopper was measured. This measurement was taken as the GT (Fig. 1). Once all the sites were registered, the abutment was retired and the operator proceeded to evaluate the GT with the next method.

2) Tension free caliper (Iwanson DP 720, Italy) (TFC). Before starting, it was modified by cutting the spring to eliminate the tension in order to avoid excessive pressure on the soft tissue (Fig. 2). Thickness was then determined with the caliper at the points marked at 1, 2 and 3mm apical to the gingival margin. When the measurements with this method were finished, the operator started with the last procedure.

3) Direct visualization measurements (DV) were done with a periodontal probe graduated in 1 mm increments (CPC-15 periodontal probe, Hu-Friedy, Leinmen, Germany). These measurements were taken directly on an incision made in the central axis of the implant (Fig. 3). The direct measurement of the thickness with the periodontal probe was considered for the authors as the reference standard.

-Statistical analysis

The subject (animal) was the unit for the basic statistical analysis. Means and standard deviation were calculated for the gingival tissue thickness. Data were expressed as medians and 95% confidence intervals. Normal distribution of continuous variables studied by the Kolmogorov-Smirnov test was not confirmed; therefore data were compared using nonparametric analysis. Wilcoxon signed rank test was used to assess differences in the GT evaluated by direct measurements (gold standard) and by two other measurement systems. Paired analysis using the same statistical test were performed after stratifying the samples according to thickness (<1 mm; 1-2 and ≥2 mm). Alpha error was set at *p* < 0.05.

## Results

The GT of 108 implants placed in 27 mandibles from female pigs was studied between February 2013 and June 2014, after application of the study criteria described before. A total of twelve measurements per specimen were made at 1mm, 2 and 3mm apical to the central aspect of the periimplant margin (soft tissue height incremental areas) of each implant, where soft tissue mean thicknesses obtained with the reference standard (DV) were 1.15±0.49 mm, 1.42±0.65 mm and 1.54±0.76 mm respectively.

-Test Results.

 Table 1 summarizes the descriptive data recorded for the GT measurements and the reference standard.

**Table 1:**
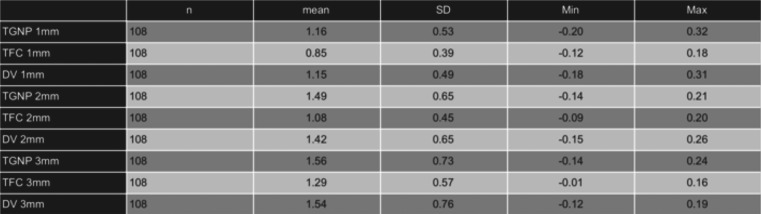
GT mean, standard deviation (SD), minimum (Min) and maximum (Max) values in mm for TGNP, TC and DV methods.

According to the results 59% of the measurements were classified as thin biotype and 41% as thick biotype. With the TGNP, the thin biotype was correctly assessed in 84% of the sites and the thick biotype in 76%. TFC was successful in assessing the thin biotype in 86% of the situations, in contrast, for the thick biotype the percentage of hit was 0%.

 Table 2 displays the Wilcoxon analysis for the accuracy data, which reveal statistically significant differences between TFC and DV (reference standard) at 1mm (*p*<0.001), 2mm (*p*<0.001), and 3mm (*p*<0.005) from the gingival margin. No significant differences were observed between the TGNP method and the reference standard (DV) at none of the measured areas.

**Table 2:**
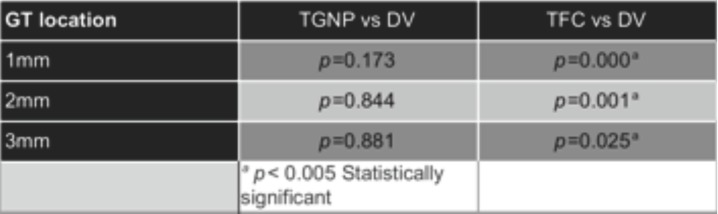
Wilcoxon test for paired samples comparing the tested methods with the reference standard.

## Discussion

This *in vitro* model was designed to determine the accuracy of peri-implant mucosa thickness by measuring it in the midfacial aspect of 108 implants placed in 27 animal mandibles. Differences between methods were detected, finding TGNP more accurate than TFC.

The invasive methods of assessing GT using an injection needle or a probe have been used traditionally by many authors (14,29–31). In 2003, Kan *et al.* (18) introduced a non invasive method which was based on the transparency of periodontal probe. It categorized the biotype as thin if the underlying of the periodontal probe was visible through the gingival or thick if not. This method has been widely used for biotype discrimination (7,10,32) and authors have considered it as a gold standard (33) even though it has been demonstrated that less than the 50% of biotypes are correctly assessed by experienced dentists (3).

 TGNP using an injection needle or an endodontic tool with a silicone limiter seems to be accepted as an accurate method despite it has not been scientifically validated for that purpose (13,34,35). Our results confirm that it is an accurate system for GT evaluation as no differences (*p*> 0.005) with the measurements obtain by DV measurements (gold standard) were observed neither at 1, 2 or 3mm points measured from gingival margin. These results indicate that GT assessment with TGNP is a reliable method no matter the thickness of the study area. If we consider the classification of Claffey *et al.* (14) (thin <1.5mm; thick≥2mm), the thin biotype was correctly assessed in 84% of the sites measured, and the thick biotype in 76%.

 When comparing this method with the Computed tomography (CT) a strong correlation between the two procedures could not be found (34). The authors concluded that CT is not as reliable as needle probing as it seems to overestimate the true thickness in areas with thin gingiva. However, the CBCT has been described as a useful method to assess palatal mucosal thickness (17,25) and for biotype classification (26). By contrast, Fu *et al.* (36) stated that CBCT provides accurate measurements of soft tissue thickness. They concluded that it is a more objective method to define the thickness of soft tissues than direct measurements.

Some authors (21,37,38) have proposed the use of a specially-designed ultrasonic dental system as a diagnostic tool for gingival thickness determination. Only one study (21) have compared the ultrasonic device with the transgingival method in an *in vitro* model, concluding that some errors are associated with the invasive technique and recommending the ultrasound method for non invasive GT assessment.

Our results are in consonant with some authors that affirm that even though the CT methods and ultrasonic devices are suitable and painless techniques for obtaining GT (23), the unavailability(39) and high costs(26) associated with these systems are disadvantages to take into consideration. Simple measurements performed with a periodontal probe are often part of routine diagnostics, which are carried out quickly without special appliances or preparations (13).

The use of a TFC for direct gingival thickness measurements has also been studied by many authors (6,22,36). Kan *et al.* (6) even considered it as a gold standard to validate other methods. However, the caliper has not been scientifically evaluated for this purpose and our results found it not reliable for thickness evaluation as significant differences were observed (*p*<0.001) with respect to our reference standard. Especially when assessing the thick biotype, the caliper failed in 100% of the cases, while for the thin biotype failed just 14% of the times. This lack of accuracy for assessing thick biotypes may be due to a compression of the soft tissues, suspecting that the spring cut of the caliper could not be valid for eliminating the tension.

Besides the inherent limitations of an *in vitro* model, the major weakness of the study is the lack of consensus of a precise definition for thick and thin biotype. The lack of studies about reliability and accuracy of biotype assessment makes difficult to compare the results of this research, therefore the external validity of our study is limited.

Within the limitations of this *in vitro* study, the present data support the following conclusions.

(i) The transgingival needle probing is an accurate method for gingival thicknesses measurements at different vertical levels from the margin.

(ii) The spring modified caliper is not valid for gingival biotype determination, especially for thick biotype, where it tends to infra-measure the real thickness of the mucosa.
